# The Forest for the Trees: A Systems Approach to Human Health Research

**DOI:** 10.1289/ehp.10373

**Published:** 2007-06-28

**Authors:** Julia M. Gohlke, Christopher J. Portier

**Affiliations:** Environmental Systems Biology Group, Laboratory of Molecular Toxicology, National Institute of Environmental Health Sciences, National Institutes of Health, Department of Health and Human Services, Research Triangle Park, North Carolina, USA

**Keywords:** public health, risk assessment, systems biology

## Abstract

We explore the relationship between current research directions in human health and environmental and public health policy. Specifically, we suggest there is a link between the continuing emphasis in biomedical research on individualized, therapeutic solutions to human disease and the increased reliance on individual choice in response to environmental and/or public health threats. We suggest that continued research emphasis on these traditional approaches to the exclusion of other approaches will impede the discovery of important breakthroughs in human health research necessary to understand the emerging diseases of today. We recommend redirecting research programs to interdisciplinary and population-focused research that would support a systems approach to fully identifying the environmental factors that contribute to disease burden. Such an approach would be able to address the interactions between the social, ecological, and physical aspects of our environment and explicitly include these in the evaluation and management of health risks from environmental exposures.

The current generation of children in many countries have a shorter life expectancy than their parents’ generation, mainly due to changing sociopolitical systems and infectious diseases such as AIDS (World Health Organization 2006). Furthermore, the epidemiological transition that has occurred in developed countries leading to the modern rise in obesity, heart disease, type 2 diabetes, autoimmune disorders (Gillespie et al. 2004; Tedeschi and Airaghi 2006), and certain types of cancers (Dinse et al. 1999) has led to predictions of decreased life expectancy for future generations (Olshansky et al. 2005). This phenomenon cannot be explained by changes in human genetics because the time frame in which they have arisen or accelerated has hardly crossed a generation (Lopez and Murray 1998). Therefore, the sharp rise in these diseases can be attributed to recent changes in our environment, defined here as encompassing social, ecological, and physical components. Logically, identifying the environmental factors that are driving these increases should be a major focus of human health research. Unfortunately, this is not the case. Our continuing emphasis on individualized, therapeutic solutions in human health research has far-reaching implications for environmental and public health policy. We offer an alternative, systems-based framework to direct human health research integrating physical, ecological, and social factors with individual aspects.

Starting with Renee Dubos in the 1960s (Dubos et al. 2005) and continuing to present day (Anderson 2004; Cornish-Bowden 2006; Ebi and Gamble 2005; Rose 2001; Schwartz et al. 2006; Toscano and Oehlke 2005; Wing 2003), it has been recognized that a reductionist approach is not sufficient for predicting factors affecting human health, yet current human health research has continued to focus heavily on the biochemical processes causing and modifying specific disease states in the individual, rather than critical analyses of the environmental determinants of health. This focus is evident in analyses of citation indices (Boyack et al. 2005; Ioannidis 2006), where productivity, connectivity, and the role of high-impact multidisciplinary journals has been evaluated. For example, basic biomedical research fields—particularly biochemistry, neuro-science, immunology, cancer biology, and microbiology—have higher citation densities and higher publication rates in the highest-impact multidisciplinary journals, namely *Nature* and *Science*, when compared with interdisciplinary or population-focused research fields such as epidemiology, social sciences, and public health. In the United States, this result is not surprising considering that > 50% of National Institutes of Health (NIH)–supported grants have principal investigators at medical schools, traditionally focused on addressing disease paradigms and therapeutic solutions, whereas approximately 2% of NIH-supported grants have principal investigators in schools of public health, traditionally focused on addressing population risks (NIH 2005). Finally, the most cited medical research is increasingly funded by industry (Patsopoulos et al. 2006), highlighting the impact of current market forces, which provide large financial incentives in the search for therapeutic solutions. In contrast, complex behavioral interventions are not easily patented, so preventive research relies almost exclusively on public and nonprofit funding (Delaney 2006). The result is a research community that is very productive in medical areas dedicated to searching for therapeutic solutions for individuals with particular diseases, but limited in areas of research identifying preventive measures.

Our reliance on basic biomedical research approaches in human health research has undoubtedly shaped current policy decisions. Hence Paul Weiss’s words, “It’s one thing not to see the forest for the trees, but then to go on to deny the reality of the forest is a more serious matter” (Weiss 1969), are particularly salient. For example, environmental health decisions are driven predominantly by single-pollutant risk assessments, generally supported by scientific research in inbred rodent models exposed to single chemicals [National Research Council (NRC) 1983; U.S. Environmental Protection Agency (EPA) 2002]. This has led to a focus on individual exposures and individual risks, promoting unusual solutions to environmentally mediated health outcomes, whose primary effect is at the population and ecosystem level.

Global methylmercury (MeHg) contamination of the aquatic ecosystem provides a particularly salient example of how our individual focus on health has affected our policies on environmentally mediated population health. To avoid adverse health effects, current U.S. EPA, Food and Drug Administration (FDA), and Institute of Medicine (IOM) recommendations suggest we minimize our exposure to MeHg by choosing, as individuals, to limit our consumption of highly contaminated species of fish, while still eating other less-contaminated species to receive the well-known nutritional benefits of fish consumption (FDA 2004; IOM 2007; U.S. EPA 1997). However, for a large portion of the world’s population, there is no real choice, because some populations have a traditional diet high in particular species of fish readily available to them and face the immediate risk of malnutrition or starvation, outweighing the risk posed by MeHg (McMichael and Butler 2005; Passos et al. 2003; Tuomisto et al. 2004). Furthermore, the suggestion that simply altering patterns of fish consumption is a viable solution neglects systemwide effects on resource depletion, pollution, and environmental destruction from overfishing (Sala and Knowlton 2006). This example suggests that, by not taking into account social and economic forces, our focus on personal choice and therapeutic fixes is failing to provide long-term solutions and is inadequate for protecting a large fraction of the global population. Alternatively, could we improve our health through a more integrated examination of the primary causes behind environmentally mediated diseases?

Our reliance on carbon-based energy, particularly coal-fired power plants, accounts for two-thirds of mercury emissions globally (Pacyna et al. 2006), making this the major determinant of MeHg in the global ecosystem, and hence the major determinant of MeHg in the fish we eat. In addition to mercury contamination, carbon-based energy increases our exposure to several air pollutants linked to increases in both immune-based diseases and cardiovascular diseases (Luke et al. 2006). Furthermore, carbon-based energy is the key determinant of global climate change with far-reaching impacts on infectious diseases, malnutrition, freshwater supplies, and heat-related mortality (McMichael et al. 2006; Patz et al. 2005). Finally, carbon-based energy is a key driver in the planning and development of our built environment, which is linked to a host of emergent diseases related to decreased physical activity and the obesity epidemic (Corburn 2004; Saelens et al. 2003). This suggests that a health policy that addresses risks associated with carbon-based energy may be much more effective for reducing disease worldwide than current recommendations of altering fish consumption. In addition, this example clearly highlights the inadequacy of the single chemical exposure/single endpoint risk assessment to develop robust health policies. To effectively evaluate the relative importance of proximal and distal upstream factors affecting environmentally mediated diseases and to compare the downstream effects of possible solutions, we propose a systems approach to health assessment that specifically evaluates the linkages between our societal choices, our environment, and our health.

Currently, growth of systems-based approaches in health research is most evident at the molecular level, with an increased collaboration between molecular biologists and computer modelers (Ideker 2004; Kritikou et al. 2006). Though the overall concept originates in physiology and is far from novel, the present goal of these methods is progression to a cellular-level systems understanding, then to the organ level, and eventually to the organism level (Kitano 2002), building networks of interactions between molecules, cells, tissues, and organs to form a predictive view of an individual organism (Figure 1). Predicting molecular-level processes, taking advantage of high-throughput technologies developed at the molecular level (chIP on chip, microarrays, proteomics), is clearly a rich area in current human health research. Although it is evident that this research is having critical impacts on defining therapeutic solutions to disease by identifying specific genetic components and/or molecular targets, these bottom-up approaches minimize complex environmental influences as determinants of health. Progression of systems biology will depend on the parallel development of top-down approaches, to identify essential environmental systems, link components within these systems, and quantitate impacts on human health (Figure 2).

The framework proposed in Figure 2 incorporates levels of organization beyond the individual, including effects of the social environment, the ecosystem environment, the physical environment, and the extraterrestrial environment, all of which play a central role in the health of the individual human being. Within this proposed framework, the social environment includes all aspects of interaction within our species (e.g., government and politics, economy, industry, built environment, community, family), whereas the ecosystem environment includes interactions with all other forms of life on earth. Most important, our ecosystem is our food supply, but it also serves as a reservoir for vectors of infectious diseases, and is the source of numerous therapeutic interventions. Our physical environment includes all nonliving aspects of the earth, such as water, air, mineral, climate, natural disasters, and previously living organisms (e.g., coal). Finally, extraterrestrial environmental factors include all planetary effects, such as sunlight and gravity, which alters health directly (e.g., skin cancer), or indirectly through interaction with physical-, ecosystem-, and social system–level effects. This figure is intended to highlight the importance of interactions between these three environmental systems in determining the health status of an individual.

The fundamental challenge for implementation of this approach will be the integration of computational biology, evolutionary, and ecosystem-based approaches, found at the intersection of more traditional mathematics and biology departments, into biomedical and public health research, as others have noted (Koopman and Lynch 1999; Levins and Lopez 1999; McMichael 1999). The use of health impact assessments in community design and public policy evaluation is an example of an initial step toward implementing a systems approach to human health (Cole et al. 2005), yet more widespread use and subsequent further development of this approach is needed. For example, the application of network theory in biology and economics will need to move beyond genomic systems and profit/loss, respectively, to incorporate environmental networks. In addition, temporal aspects of network relationships, on the geological, evolutionary, and generational scale, are an important component that must be addressed to create sustainable health.

Our genome is the product of environmental pressures that have molded the genetic makeup of species throughout evolution. Our remarkable adaptability is attributed partly to our capacity to modify our environment; yet how much can we transform the environment before it is detrimental to the survival of our species? At what point will our capacity to adapt be overwhelmed by the rate at which we are changing our environment? It is only through a fully integrated systems approach, explicitly linking research on the human system with research on the environmental system and coupled with an effective strategy of applying this research, that we can hope to make the scientifically sound and sustainable environmental decisions that are critically needed now.

## Figures and Tables

**Figure 1:**
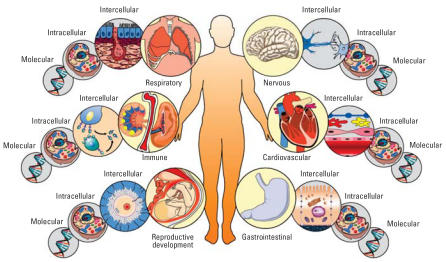
Systems biology framework for the individual. Current systems biology methodologies take advantage of high-throughput data generated at the molecular level in the hope of one day translating these maps of molecular interactions into cellular-level responses, then intercellular responses, and finally to an organ-level response. The interconnections between organ systems will need to be elucidated to understand an organism-level system.

**Figure 2:**
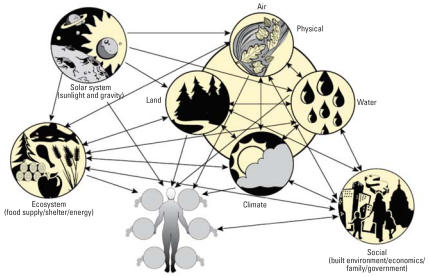
Interaction network between our environment and our health. Human health is determined not only by various molecular, cellular, and organ system–level systems, but by our environment, including social (all interaction within our species), ecosystem (all interactions with other life on earth), physical (all interactions with nonliving components of the earth), and extraterrestrial (planetary position, energy from sun, gravity). Arrows indicate major highways of interaction determining potential routes of global or local changes within these systems. All systems have the potential to affect the individual’s health status.
